# A call for better understanding of social media in surveillance and management of noncommunicable diseases

**DOI:** 10.1186/s12961-021-00683-4

**Published:** 2021-02-10

**Authors:** Chi-Wai Lui, Zaimin Wang, Ning Wang, Gabriel Milinovich, Hang Ding, Kerrie Mengersen, Hilary Bambrick, Wenbiao Hu

**Affiliations:** 1 School of Public Health, The University of Queensland, Brisbane, QLD Australia; 2 Centre for Chronic Disease, School of Clinical Medicine, The University of Queensland, Brisbane, QLD Australia; 3 School of Public Health and Social Work, Queensland University of Technology, Brisbane, QLD Australia; 4 RECOVER Injury Research Centre, Faculty of Health and Behavioural Sciences, The University of Queensland, Brisbane, QLD 4059 Australia; 5 ARC Centre of Excellence for the Mathematical and Statistical Frontiers, School of Mathematical Sciences, Queensland University of Technology, Brisbane, QLD Australia

**Keywords:** Social media, Noncommunicable diseases, Surveillance, Management

## Abstract

Using social media for health purposes has attracted much attention over the past decade. Given the challenges of population ageing and changes in national health profile and disease patterns following the epidemiologic transition, researchers and policy-makers should pay attention to the potential of social media in chronic disease surveillance, management and support. This commentary overviews the evidence base for this inquiry and outlines the key challenges to research laying ahead. The authors provide concrete suggestions and recommendations for developing a research agenda to guide future investigation and action on this topic.

## Main text

We are living in a global digital age and social media has become embedded in almost every aspect of our daily life. Social media, as Nicole Ellison and Danah Boyd [1] explained, is ‘a networked communication platform in which participants (1) have *uniquely identifiable profiles* that consist of user-supplied content, content provided by other users, and/or system-level data; (2) can *publicly articulate connections* that can be viewed and traversed by others; and (3) can consume, produce, and/or interact with *streams of user-generated content* provided by their connections on the site’ (emphasis in original). With the proliferation of the Internet, social media has developed into a primary medium from which people seek or share health information and experiences, or search for support [2, 3].

Over the past decades, health professionals and researchers have learned of the value and potential of social media in data gathering, patient education and engagement [4, 5]. In particular, the analytics of data produced by social network sites or groups has been considered an effective and low-cost approach to complement traditional public health surveillance approaches [6]. However, many of these discussions have centred on acute or epidemic-prone communicable diseases and there is a scarcity of literature on the role of social media in the surveillance of chronic illness. A systematic review of applying Internet-based sources for public health surveillance found that only about 11% (17 out of 162) of the studies on this topic focus on chronic disease [7].

Given population ageing and changes in health profiles and disease patterns, there is a shift in global health system focus from treatment to care and management of long-term conditions. The epidemiologic transition to noncommunicable diseases calls for a paradigm shift in healthcare in favour of service integration, community care, partnership and teamwork, and patient as a principal caretaker [8]. In 2011, as a response to the emergence of chronic disease pandemics the United Nations General Assembly made a joint declaration on preventing and controlling noncommunicable diseases worldwide. In such a context, this commentary overviews the existing evidence in using social media data or platform for tackling chronic diseases. It aims to raise attention of health professionals and policy-makers on the potential of these approaches in global health, identify issues requiring further investigation, and suggest areas for implementation to address critical chronic health issues.

### Potentials of social media for chronic disease surveillance and management

There is evidence that social media is an important source for patients of chronic disease to seek health information [2, 9, 10]. But for many people living with a long-term condition, social media platforms are more than just an information vending machine. They also act as a forum for the exchange of lived experience around chronic disease, a source of emotional and instrumental support, or a place to comment on or recommend care services [11–14]. These diverse uses of social media play crucial roles in the self-management of chronic diseases. A systematic review found that online communities have several key functions in the management of chronic conditions including work, identity, social support and connectivity, experiential knowledge sharing, collective voice and mobilization [15]. Further to this, identity, flexibility, structure, narration and adaptation have been identified in another review as the affordances underpinning the effects of social media in chronic disease management [16].

Although misinformation about health can spread rapidly through social media, attitudes and practices regarding chronic illness shared within online platforms also create a valuable resource for public health surveillance and monitoring [17]. Research findings confirmed that social media users openly discussed a wide variety of health practices, dietary and lifestyle behaviours in real time and these exchanges are valuable for identifying populations who have or are at risk of developing noncommunicable disease [18–20]. A recent review of applications of big data analytics to chronic disease management found evidence of using social media data to determine risk factors and patient readmission, increase diagnostic accuracy and patient outcome, achieve better treatment guidance and cost reduction [21]. Applying big data analytics to address chronic disease is still much in its infancy but has shown significant promise.

Researchers and policy-makers have learned of the potential of social media tools and mobile health apps to be incorporated into disease management and education for lifestyle modification [22, 23]. Existing evidence suggests that using social media improves care and health outcomes of chronic disease patients and that reports of adverse events or harm are rare [16]. A recent meta-analysis of 53 systematic reviews of online interventions to support self-management of chronic conditions confirmed that such intervention is a viable and safe option for delivery of support in long-term conditions, especially heart failure and type 2 diabetes mellitus [24]. Using social media in supporting the psychosocial management of chronic conditions is an area of particular optimism [15, 16]. The benefits of web-based interventions for treating depression in people with chronic illness have been established [25]. However, there is an urgent need for evidence of social media as a means of psychosocial support for other chronic diseases, such as arthritis, back pain and cancer, which constitute a major portion of global disease burden.

Whilst there are descriptive accounts of online communities of people living with chronic disease, little is known of the properties of these communities and how they function and evolve over time [26, 27]. Given that social media and adjacent technologies are a complex and fast-changing phenomenon, there is a need for continuous research on the dimensions of affordances, affective forces and agency provided in different online platforms and the complex ways participants are engaging with each other in these sites and contributing to health information or disease-management practice.

## Conclusions: challenges ahead

Social media as a platform for open communication and source of data has considerable potential for understanding how people perceive and manage chronic conditions in everyday life. The growing popularity of social media worldwide as a source for health information and discussion presents an unprecedented opportunity for researchers to collect data and connect with people living with chronic conditions. The time has come for developing a public health research agenda to guide investigation on this topic.

In Australia, the Federal Government invested over $200 million in 2018 to support the development of digital health initiatives via the National Health and Medical Research Council Centre of Research Excellence in Digital Health (https://digitalhealth.edu.au/) and the latter will play a key role in facilitating and supporting multidisciplinary and collaborative research in chronic disease in years to come. Specifically, we call for targeted investigation into using social media for chronic disease surveillance and management; and to advance research, we recommend the research community to focus on:

- developing integrated approaches to collecting and analysing social media data from both traditional and newly emerged platforms to gain an understanding of experiences across the entire chronic disease continuum;
- designing novel methods for extracting signals from social media data to identify upstream risk factors;
- developing advanced spatiotemporal statistical models based on social media data to monitor trends of noncommunicable diseases or behaviour patterns;
- developing an understanding of social media strategies for the delivery of support and intervention for improving self-management of chronic conditions;
- exploring evidence-based approaches to utilise social media networks and technologies to elicit beneficial behaviour modification, improve health promotion, and fight bad health advice or misinformation;
- identifying legal and ethical issues in and constructing guidelines for using social media data for the prevention and control of chronic diseases; and
- developing a policy framework for evaluating the quality and validity of publicly available health information and lifestyle recommendations in social media.

## Data Availability

Not applicable.
